# Retrospective Study of Diabetic Retinopathy and Macular Edema in Southern Part of Bangladesh

**DOI:** 10.1155/2024/8437947

**Published:** 2024-06-06

**Authors:** Md. Asif Hasan, Sheikh Md. Rabiul Islam, Md. Arif Hayat Khan Pathan

**Affiliations:** ^1^Department of Biomedical Engineering, Khulna University of Engineering and Technology, Khulna 9203, Bangladesh; ^2^Department of Electronics and Communication Engineering, Khulna University of Engineering and Technology, Khulna 9203, Bangladesh; ^3^Department of Vitreo-Retina, Ispahani Islamia Eye Institute and Hospital, Dhaka, Bangladesh

## Abstract

**Background:**

Diabetic mellitus is a vision-threatening disease because it causes diabetic retinopathy worldwide. The main focus of this research is to determine the prevalence and assess the visual outcome in diabetic retinopathy and macular edema patients by injecting Bevacizumab clinically.

**Methods:**

This hospital-based trial case was conducted in Khulna BNSB Eye Hospital, Bangladesh. This study is based on a prospective cohort with a population of macular edema in 41 eyes of 25 diabetic patients, of whom 94 were diagnosed with diabetic retinopathy in 320 type 2 diabetes mellitus patients. The treating physician inserts 1.25 mg (0.05 ml) into the patient's eye. We have used optical coherence tomography (OCT) and colour fundus photography (CFP) for an eye check performed on all patients before and after the injection of Bevacizumab. The method results analyze the effects of the technique using IBM SPSS 25.

**Results:**

The study population selected 25 patients with 41 eyes for clinical investigation by injection of Bevacizumab. The net effects of this study on five eyes with macular edema were entirely resolved. It was BCVA from 6/6 to 6/9. The 29 eyes were partially resolved, which is called improved visual acuity, and BCVA was 6/12 to 6/60. In the case of seven eyes, we found that the vision did not change before or after the Avastin injection. No change was seen in seven eyes of macular edema due to the effects of the Avastin injection before and after.

**Conclusions:**

In clinical trial-based research, Bevacizumab (Avastin) is best effective for diabetic retinopathy (DR) and diabetic macular edema (DME) patients.

## 1. Introduction

One of the leading causes of blindness in diabetic people is diabetes mellitus. For people with type-2 diabetes mellitus (DM) who have had the condition for a long time, diabetic retinopathy (DR) is the leading cause of concern [1–5]. Patients with diabetes mellitus are often ignorant of diabetic retinopathy, and managing their condition over a number of years is now the primary source of suffering [6–8]. Diabetic retinopathy (DR) and blindness can be avoided if blood glucose levels are monitored often and kept within the recommended range [9].

Patients with diabetes mellitus must eventually acquire diabetic retinopathy due to their uncontrolled blood sugar levels. Patients with diabetic macular edema will experience visual loss. With macular edema or diabetic macular edema (DME), they can be treated as PDR and NPDR [7]. The primary objective of preventing blindness can be lessened globally if diabetes mellitus patients are identified early and given the appropriate treatment [10–12].

In DR, a new blood vessel that the patient has created is the source of the blood leak. The inner retinal barrier's collapse, which is mediated by a number of growth factors, is the primary cause of retinal injury. Macular edema is caused by vascular endothelial growth factors (VEGF), which enhance vascular permeability and blood vessel leakage [13, 14]. In the pathophysiology of diabetic macular edema and diabetic retinopathy, anti-VEGF medications may be a therapeutic option. Anti-VEGF has been linked to diabetic macular edema and diabetic retinopathy, according to recent research [12–21]. They demonstrated the remarkable efficacy of intravitreal bevacizumab injections in the treatment of diabetic macular edema and diabetic retinopathy. Anti-VEGF medications are available in a number of forms, including Aflibercept, Ranibizumab, and Bevacizumab (Avastin). In this investigation, intravitreal injections of bevacizumab (Avastin) were utilized. All study populations can afford it because of its affordability. We discovered that other anti-VEGF outcomes for the regression of diabetic macular edema are unknown; more research is still needed to determine whether improved corrected visual acuity is beneficial for subsequent investigations.

A humanized anti-VEGF antibody is bevacizumab, also known as Avastin. It suppresses all active VEGF forms while binding all VEGF subtypes. Treatment options for bevacizumab include choroidal neovascularization, iris neovascularization, macular edema from vitreous haemorrhage, and metastatic colorectal cancer. This bevacizumab demonstrated numerous advantages for diffuse diabetic macular edema (Avastin) injection in the research field [13–21]. This investigation's primary goals were the following:

- To evaluate the analysis of intravitreal Bevacizumab therapy to reduce diabetic macular edema
- To examine and contrast the overall improvement in vision loss from DME prior to and following intravitreal Bevacizumab treatment

## 2. Research Design

### 2.1. Study Design

This research is a hospital-based cohort study that aimed to measure the effectiveness of injection of Bevacizumab (Avastin) in treatment procedures for diabetic retinopathy, not only PDR but also moderate and severe NPDR with CSME for DR, among patients with type 2 diabetic mellitus patients in the city of Khulna, Bangladesh.

### 2.2. Study Area

This study was conducted at Khulna BNSB Eye Hospital, Bangladesh.

### 2.3. Study Population

**Figure 1:**
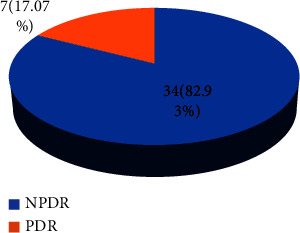
Number of PDR and NPDR with macular edema patients.

All Type 2 diabetic patients with diabetic retinopathy (DR) who received care at Khulna BNSB Eye Hospital were included in this study. The consent form and description of data collection were provided to the patients in this study by the volunteers. The interviewer read the consent if the subject had vision loss or was illiterate and could not understand the data declaration. As shown in Figure 1, this study is based on a prospective cohort of 25 diabetic individuals who had macular edema in 41 of their eyes. Each of the groups had macula edema, along with NPDR and PDR. Every patient received intravitreal bevacizumab injection treatment after being chosen from the hospital's outpatient department (OPD).

#### 2.3.1. Sample Size Calculation

We selected the data collection as a feasible sample size for this research.

#### 2.3.2. Sample Selection Technique

The data collection used a simple random sampling technique in this research.

### 2.4. Data Collection

The protocol for collecting data includes best-corrected visual acuity (BCVA) using the Snell Chart, slit-lamp bio-microscopy for dilated fundus examination with a 90D lens, fluorescence fundus angiography, and lab tests to check their glycemic control. We have considered follow-up for four-time visits for each patient for BCVA and macular oedema in each month.

### 2.5. Exclusion and Inclusion Criteria

Before receiving the intravitreal bevacizumab injection, the patients should undergo a check-up; the study excluded patients with conditions such as hypertension, myocardial infarction, cataracts, laser photocoagulation, and glaucoma. The selection criteria for patients of both genders and ages ranged from 30 to 70 years old. All of them had PDR and NPDR with diabetic macular oedema disease.

### 2.6. Data Collection Procedure

**Figure 2:**
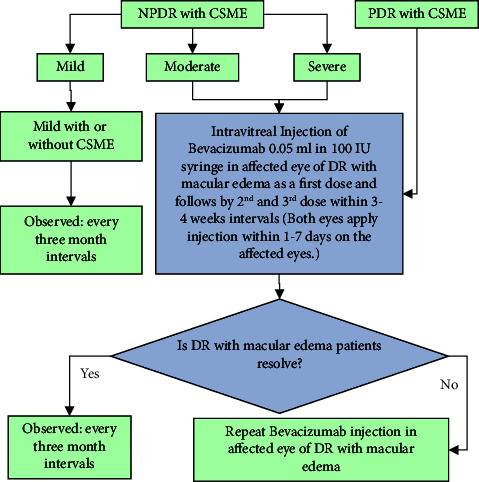
Flowchart of clinical treatment procedures for diabetic retinopathy patients.

We also subjected this study to examination procedures such as fluorescence fundus angiography (FFA) and slit-lamp inspection. The patients need to have reasonable, clear media and not have had laser photocoagulation or intravitreal eye therapy in the past.  Figure 2 depicts the study population and the treatment criteria flowchart. In this procedure, patients with PDR with CSME, severe NPDR, and moderate NPDR have received an injection of bevacizumab. We excluded patients with or without CSME who had minor NPDR and checked on them every three months.

### 2.7. Case Study

This study selected two groups of patients: NPDR with CSME with macular edema and PDR with CSME with macular edema.

#### 2.7.1. Case 1

We classified NPDR with CSME and macular edema, which includes 34 eyes in 21 patients, into three (3) groups.

- Group 1: each patient's BCVA is 6/60 or less
- Group 2: each patient's BCVA is between 6/36 and 6/24
- Group 3: each patient's BCVA is 6/18 or better

#### 2.7.2. Case 2

We again divided the PDR with CSME and macular edema, which includes seven (7) eyes in four (4) patients, into two (2) groups.

- Group 1: each patient's BCVA is less than 6/60
- Group 2: each patient's BCVA is 6/60 or better

### 2.8. Treatment Protocols

#### 2.8.1. First Sequel Procedures

(i) Using a standard aseptic technique, we inject the available solution (100 mg/4 mL) into 1-mL tuberculin syringes containing 0.05 mL before inserting it into the patient's eyes. We stored it under sterile conditions at 4°C in the refrigerator.
(ii) The treating physician inserts a 1.25-mg (0.05 ml) injection into the patient's eye. We applied Moxifloxacin eye drops four times daily before the injections and continued for one week.
(iii) All aspects of techniques are as follows:
  1. We applied scrubbing with 5% povidone-iodine on the ocular surface and topical anaesthetic.
  2. We used an eyelid speculum to stabilize the eyelid.
  3. We injected 1.25 mg (0.05 ml) of Bevacizumab 3.5–4 mm posterior to the limbus using a 30-gauge needle.
(iv) We followed up on the intraocular pressure and retinal artery perfusion and administered topical antibiotics for seven days for the individual. In cases of both eyes, we consider injections days after to 7 days apart.
(v) We repeated the above procedures at four-week intervals for three months.

#### 2.8.2. Second Sequel Procedures

- In response to the drugs individually and the FFA and BCVA examinations, the injection is followed by up to 3–6 months every month.
- We inserted an intravitreal injection of Bevacizumab 0.05 ml in a 100-IU syringe in the affected eye of DR with macular edema as a first dose, followed by 2^nd^ and 3^rd^ doses within 3-4 week intervals (both eyes apply injection within 1–7 days on the affected eyes). After insertion of the first two injections, those in the BCVA range of 6/12 who had macular edema reduced significantly. There is no need for a third injection; it was only observed in that patient within 3 months.

## 3. Data Processing and Analysis

We used IBM SPSS software version 25.0 for data entry and analysis. For descriptive statistical analysis, such as calculating the mean and standard deviation, this study used quantitative data. We used a logistic regression model and an ANOVA test with a *P* value to find correlations between DR and physiological traits.

- The International Classification of Diabetic Retinopathy and Diabetic Macular Edema [2, 6] classified DR severity into three categories: mild, moderate, and severe.
- We computed percentages and frequencies for categorical variables such as gender and best-corrected visual acuity (BCVA). We considered *P* values less than 0.05 as statistically significant.

## 4. Results of the Research Design

We collected data from a data sheet, and as Figure 1 shows, we selected 41 eyes from 25 patients for this cohort analysis. The diabetic retinopathy was the basis for the patient selection. After reviewing the pathology report, Snell chart, and FFA, we identified 21 NPDR and 4 PDR patients from the 25 total cases. There were 11 (44% females) and 14 (56% males) present. Two groups are included in the study: first, NPDR with CSME and macular edema, including 34 eyes in 21 patients; and second, PDR with CSME and macular edema, comprising 7 eyes in 4 patients.

Table 1 displays the results for the case of NPDR with CSME in patients with macular edema, which the methodology refers to as Case 1. We determine the eye's best-corrected visual acuity range using the Snell chart. We treat the eye with this measurement before administering an Avastin injection. 6/18 visual acuity in 7 eyes (20.59%), 6/24 in 11 eyes (32.36%), 6/36 in 11 eyes (32.36%), 6/60 in 3 eyes (8.81%), and <6/60 in 2 eyes (5.88%) were the results of this trial.

Table 2 displays the NPDR results for CSME patients, further segmenting them into three (3) groups according to their best-corrected visual acuity (BCVA) prior to Avastin injection. We have found 34 eyes for NPDR and divided them into three groups:

- Group 1: BCVA was 6/60 or less for 5 eyes
- Group 2: BCVA was 6/36–6/24 for 22 eyes
- Group 3: BCVA was 6/18 or better for 7 eyes

Avastin injection-related and preinjection BCVA values are contrasted in Table 3. Five of Group 1's patients, it appears, had BCVAs of 6/60 or lower. Although there is hope for one patient, the range of BCVA improved by 6/9 and now falls between 6/12 and 6/18. Two patients fall under the 6/24–6/36 moderate category. One (1) does not, however, demonstrate a sufficient improvement. These findings are potentially significant, according to this clinical cohort study (*P* < 0.0001). Between 6/36 and 6/24, a total of 22 patients were chosen for Group 2 based on BCVA. Following a three-month course of Avastin treatment, two patients experienced an improvement in visual acuity from 6/6 to 6/9, eleven patients exhibited improvement between 6/12 and 6/18, and seven patients showed partial improvement by BCVA 6/24. Nevertheless, there was no improvement in the patients' circumstances. *P* < 0.0001 indicates that these data are clinically significant. Based on the BCVA range and other physiological variables, we discovered that seven patients were included in Group 3 following three months of Avastin treatment. With the content of 6/18, the group's BCVA range has been clinically improved, whereas there were no patients in BCVA on 6/6, 6/9 got two patients, and 6/12 got four patients. On the other hand, one (1) patient has shown no clinical improvement. The data analysis by SPSS showed that it was hypothetically significant in this cohort study.

Twenty-one NPDR patients—six on the right and eighteen on the left—have had their 34 eyes chosen. The baseline macular thickness measured by optical coherence tomography (OCT) and the BCVA values obtained using the Snell chart are displayed in Tables 4 and 5 before and after three months (mm) of intravitreal Avastin injection therapy. It is known that the usual range for optical coherence tomography is small (≤250 *μ*m), medium (>250–400 *μ*m), or large (>400 *μ*m) [14].  Table 4 demonstrates the considerable improvement in patient no. 36's BCVA of 6/9 and OCT macular thickness of 233 mm for the right eye. This represents the 16 right-eye NPDR patients' best-corrected visual acuity. In patient number 10, the OCT macular thickness was 479 mm before and 454 mm after the Avastin injection. The BCVA was 6/60 both before and after the injection. After receiving Avastin injections for the right eye once a month for three months, the patient's condition did not get better. In contrast, patient number five's prior vision was 6/60. After three months of treatment, the OCT thickness increased from 308 mm to 303 mm, and the vision improved by 6/36. Prior to and following three months of intravitreal Avastin injection, patient numbers 35 and 43 both showed sequential improvement (BCVA 6/36 to BCVA 6/18). For the 35 patients, the macular OCT range was 327 mm to 277 mm, and for the 43 patients, it was 429 mm to 267 mm. The results of BCAV 6/60 to 6/18, however, were extremely clinically significant for patient no. 70, whose macular OCT ranged from 436 mm to 284 mm. Following three months of Avastin injection, the clinical status of patients 8, 28, 56, and 57 has improved somewhat.

Eighteen (18) NPDR patients with left eyes have been chosen in Table 5 for fundus and macular thickness examinations. Patients 36, 43, and 57 have obtained the highest levels of visual acuity, with VAT scores of 6/24, 6/60, and 6/18. After three months, their BCVA was 6/9, and their OCT macular thickness improved from 317 mm to 221 mm, 459 mm to 218 mm, and 364 mm to 220 mm. After three (3) months of intravitreal injection, patient number 10's final BCVA was also 6/18. The patient's baseline visual acuity test was 6/18. The baseline VAT test results for patients 8, 22, and 28 were 6/36; however, they improved to 6/24 with OCT ranges of 358 mm to 328 mm, 437 mm to 252 mm, and 462 mm to 298 mm. These have been partially found to have modest clinical significance based on this analysis. For patient number one, the final VAT was 6/36 both before and after a month of intravitreal injection; the prior VAT was less than 6/60. There are no noteworthy outcomes, yet patient number 60's before eyesight was 6/36 and their after vision was 6/36. Macular thickness on OCT measured 357 mm to 338 mm.

The intravitreal Avastin injection helped 34 eyes of 21 NPDR with CSME and DME patients. The FFA image, OCT image, and VAT test showed that the problem was fixed completely, partially, or not at all.  Table 6 shows that the injection of Avastin has completely resolved 14.7% of 5 patients, partially resolved 73.53% of 25 patients, and not resolved clinically in 11.77% of 4 patients.

In the case of PDR with CSME with macular edema patients, there were seven (7) eyes of 4 patients, which included 1 (25% male) and 3 (75% female). We treated them as before with a 3-month intravitreal injection, resulting in BCVA less than 6/60 in 5 eyes (71.42%). On the other hand, Table 7 displays BCVA 6/60 for two (2) eyes (28.58%). We classified the eyes into two groups (shown in Table 8):

- Group 1: BCVA was less than 6/60 for five (5) eyes (71.42%)
- Group 2: BCVA was 6/60 for two (2) eyes (28.58%)

We also cluster or group the PDR patients in two (2) ways for three (3) months before and after injection of intravitreal Avastin and check the significance of the injection clinically, as shown in Table 9. For the first cluster, we have chosen five (5) eyes with BCVA less than 6/60 before three (3)-month injections of Avastin. One (1) eye's vision improved to 6/36 after three (3) months of Avastin injection, while four (4) eyes showed no improvement. However, we did not find any eyes shown in the range of BCVA from 6/6 to 6/24. Group 2's BCVA counted two (2) eyes. After three (3) months of Avastin injection, one eye changes its vision from 6/60 to 6/24 and 6/36. We have found that these data are clinically significant (*P* < 0.0001).

Table 10 shows the results of three (3) patients with PDR in the case of the right eye. The patient's numbers 23, 32, and 33 BCVA were 6/60 to less than 6/60 before the Avastin injection. Patient number 23 shows that BCVA changes after three (3) months of applied Avastin injection. The visual acuity was 6/24, and the OCT thickness was 363 mm to 279 mm. In the cases of patients' numbers 32 and 33, before three (3) months of the injection Avastin, vision was less than 6/60 in both cases. After three (3) months of observation, mild improvement happened in patient number 32, and it turned into 6/60 with OCT 472 mm and changed into 384 mm. On the other hand, in patient number 33, the OCT range was changed from 468 mm to 456 mm, whereas BCVA remained the same.

Four (4) patients with PDR for the left eye investigation are shown in Table 11. The BCVA of patient number 5 was 6/60 both before and after the Avastin injection; there has been no discernible change in this score. 381 mm was the OCT macular thickness; this value changed to 391 mm. Before receiving the Avastin injection, the patient's numbers 23, 32, and 33 BCVA were less than 6/60. After receiving an Avastin injection for three (3) months, patient number 23 exhibits a change in BCVA. OCT thickness measured between 326 and 273 mm, and visual acuity was 6/36. Nevertheless, patients 32 and 33 have simply altered the OCT thickness and have not shown any increase in visual acuity.

Lastly, Table 12 displays the Avastin injection outcomes for the PDR patients' seven (7) eyes. The specifics showed that there were 0 (0.0%) PDR patients whose edema had completely vanished, 3 (42.85%) PDR patients whose ocular fluid had partially disappeared, and 4 (57.15%) PDR patients whose condition had not improved.

**Figure 3:**
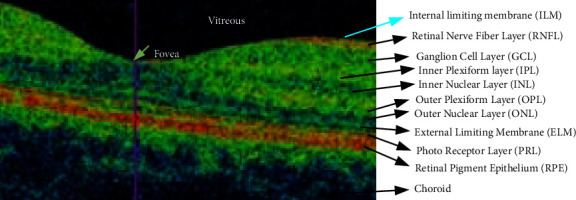
A typical OCT scan of a normal retina showing the 10 distinct layers [19].

Figure 3 displays the layers of a typical retinal OCT image. After passing through the vitreous, anterior, posterior, and corneal layers, light finally reaches the retina. The retina is composed of ten layers: the external limiting membrane (ELM), photoreceptor layer (PRL), retinal pigment epithelium (RPE), inner plexiform layer (IPL), inner nuclear layer (INL), outer plexiform layer (OPL), outer nuclear layer (ONL), retinal nerve fibre layer (RNFL), ganglion cell layer (GCL), and external limiting membrane (ILM). When someone has diabetic retinopathy with CSME, fluid builds up between the inner nuclear layer (INL) and the outer plexiform layer (OPL), which causes the fovea to form in the wrong place and vision to become blurry.

**Figure 4:**
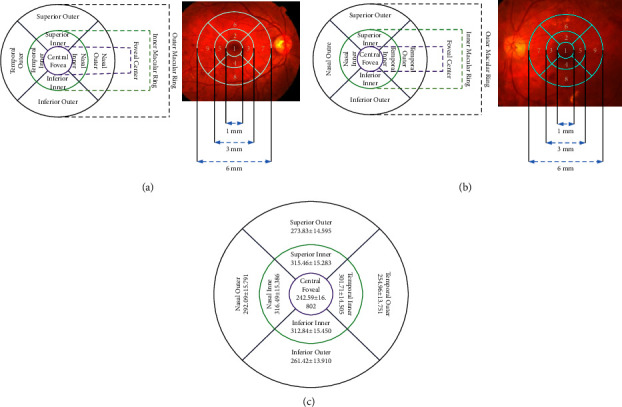
Early treatment of diabetic retinopathy study (ETDRS) grid of (a) the right eye and (b) the left eye; (c) normative data for retinal thickness in various ETDRS subfields [20].

Figures 4(a) and 4(b) depict the grids for the left and right eyes of the early-treatment diabetic retinopathy study (ETDRS). We calculate the thickness of the macula up to the internal limiting membrane of the retinal pigment epithelium. The ETDRS grid denotes the thickness and counts it from 1 to 9. Three circles make up the ETDRS grid: the outer, inner, and innermost circles. The innermost circle, further divided into four quadrants, represents the central subfield thickness of the macula. In total, this results in approximately nine sectors in the ERTDRS grids. Every section depicts the macula's thickness that we find in patient reports. Next, we use Figure 4(c) to compare it with the normative data.

There is a different colour shown in the OCT image, which indicates the following:

- The colour pink indicates the macular severe danger zone (100%)
- The light yellow, high-moderate macular danger zone (99%) is considered safe
- Green is a moderate danger zone for maculars (95%)
- Deep yellow, mild danger zone of macular thickness (5%)
- Red indicates the slightly dangerous zone of macular thickness

**Figure 5:**
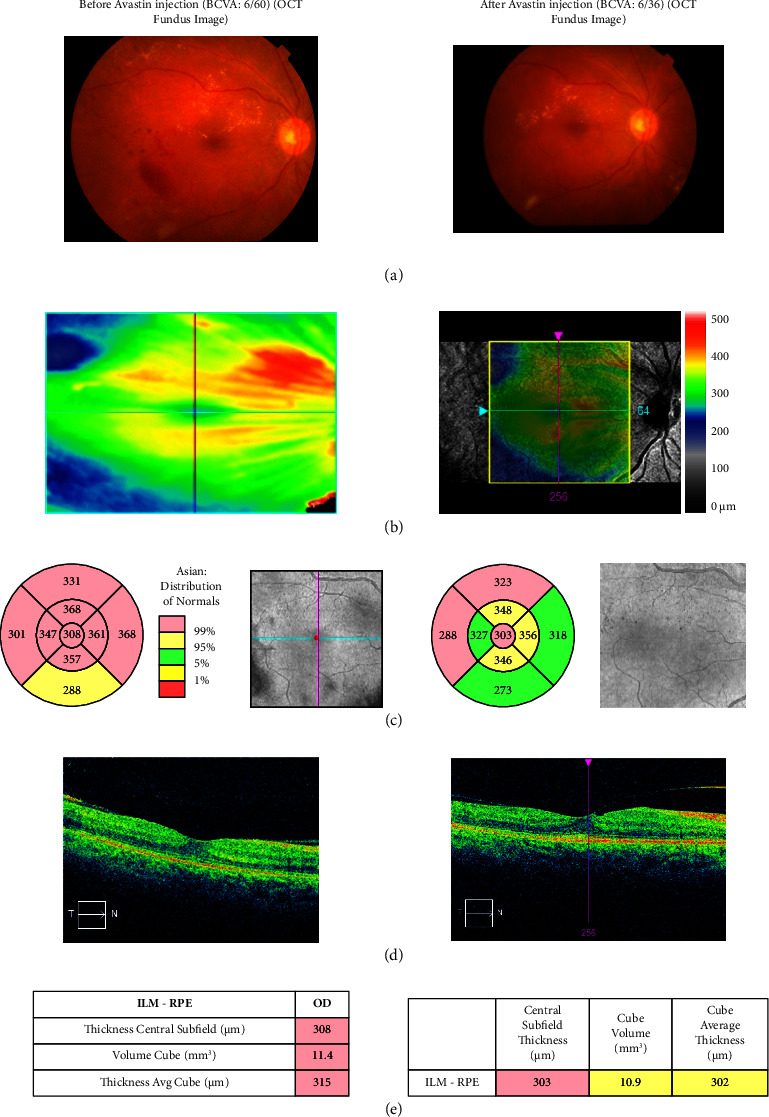
Visual outcome of NPDR (right eye) patient number 5 based on (a) CFP image, (b) OCT red free fundus image with a thickness map, (c) ETDRS grid with thickness measurements displayed in each macular sector, (d) vertical tomogram, and (e) value table. Colour codes: red: high reflectivity (white arrow); black: low reflectivity (blue arrow); green: intermediate reflectivity (magenta arrow). Normal retinal structures are labelled as red for RNFL (arrowhead) and junction of inner and outer segments of photoreceptors (PR), green for plexiform layers (magenta arrow), and blue/black for nuclear layers (blue arrow). N: nasal. T: temporal [22].

Figure 5 displays the colour fundus photography (CFP) of patient number 5, a 49-year-old woman with a 15-year history of diabetes. Prior to the administration of the injection, the patient's prior visual acuity score was 6/36. A close look at the CFP image in Figure 5(a) through the fundus revealed hard exudates and dot and blot haemorrhage as signs. On the other hand, following three months of Avastin injection, Figure 5(b) shows the decline of dot and blot haemorrhage, hard exudates, and visual outcome 6/12.

The fundus image's line scanning of the thermoscope appears in Figure 5(b). We refer to the gray areas of the fundus image as a tomogram; this is the box that solely contains the thickness profile. The macular thickness profile, also known as a macular tomogram, is represented by the horizontal (light green colour line) and vertical (pink colour line) lines in the grayscale image. To obtain distinct OCT images in different areas, this line has to be moved.

Figure 5(c) depicts the early-treatment diabetic retinopathy study (ETDRS) grid. It computes the macula's thickness up to the retinal pigment epithelium's internal limiting membrane. The thickness is measured and inputted into the ETDRS grid. There are three circles in the ETDRS grid: the inner circle, the outer circle, and the innermost circle. The innermost circle represents the macular's core subfield thickness, further dividing it into four quadrants. In total, this gives us around nine sectors in the ERTDRS grids. These are all representations of the macular thickness that we found based on patient reports. When we compare the normative data, we see that the colours are different. Yellow denotes a moderate area, red denotes a dangerous zone, and green indicates a safe zone.

Figure 5(d) shows a macular cross-hair line image in the horizontal direction (HD). The direction of the arrow in the box, pointing from temporal (T) to nasal (N), indicates the macular thickness, as captured in the image. Prior to receiving an Avastin injection, the patients' macular thickness measured 308 *μ*m, with a fluid level over the normal limit. After the injection, the macular thickness decreased to 303 *μ*m.

Figure 5(e) displays the macular characteristics of the eye. Cube volume, cube average thickness, and centre subfield thickness are its three properties. It is the centermost portion of the ETDRS grid in terms of central subfield thickness. The macular cube average thickness for the nine ETDRS grid sectors changed from 315 *μ*m to 302 *μ*m, and the macular cube volume decreased from 11.4 mm^3^ to 10.9 mm^3^. The colour-coding calculation comes next. Less than 1% of individuals display the abnormal red colour in the colour coding; the safer zones, represented by green and yellow, exhibit higher quantities in normal individuals.

**Figure 6:**
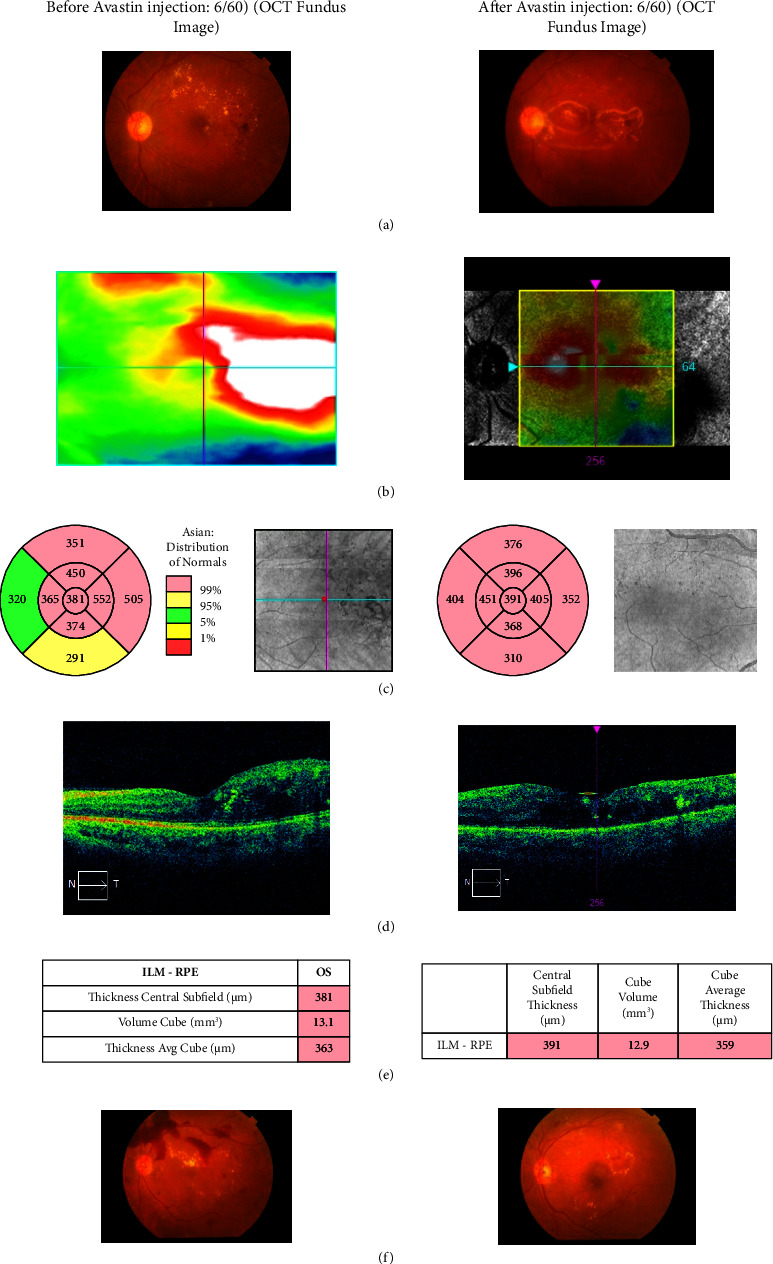
Based on CFP and OCT images, the visual outcome of PDR (left eye) patient number 23 is shown. The images include: (a) CFP images before (left side) and after (right side); (b) OCT red free fundus images with a thickness map before (left side) and after (right side); (c) ETDRS grid with thickness measurements displayed in each macular sector before (left side) and after (right side); (d) vertical tomogram before (left side) and after avastin injection (right side); and (e) value table before (left side) and after (right side)., (f) CFP images before (left side) and after (right side) of patient no. 5.

In a similar manner, as seen in Figure 6, we also looked into CFP images for PDR patients. Patients 5 and 23 are a male and female pair, aged 70 and 48, respectively. They have a combined 18-year history of diabetes under patient number 5 and 12 years under patient number 23. They had suffered an abrupt loss of eyesight, with their left eye's visual acuity being less than 6/60 for patient number 23 and 6/60 for patient number 5. The fundus examination identified the characteristics of the left eye, as seen in Figure 6(a) CFP image and Figures 6(b)–6(d) OCT images for patients nos. 5 and 23. CFP images in Figure 6(f) depict the effects of Avastin injection before and after, which are hard exudates and bleeding. CFP images (Figure 6(f)) depict the effects of avastin injection before and after, which are hard exudates and bleeding in neovascularization in the disc (NVD) and retinal neovascularization (NAV) elsewhere. Three intravitreal bevacizumab injections partially resolved these problems in one-month intervals.

**Figure 7:**
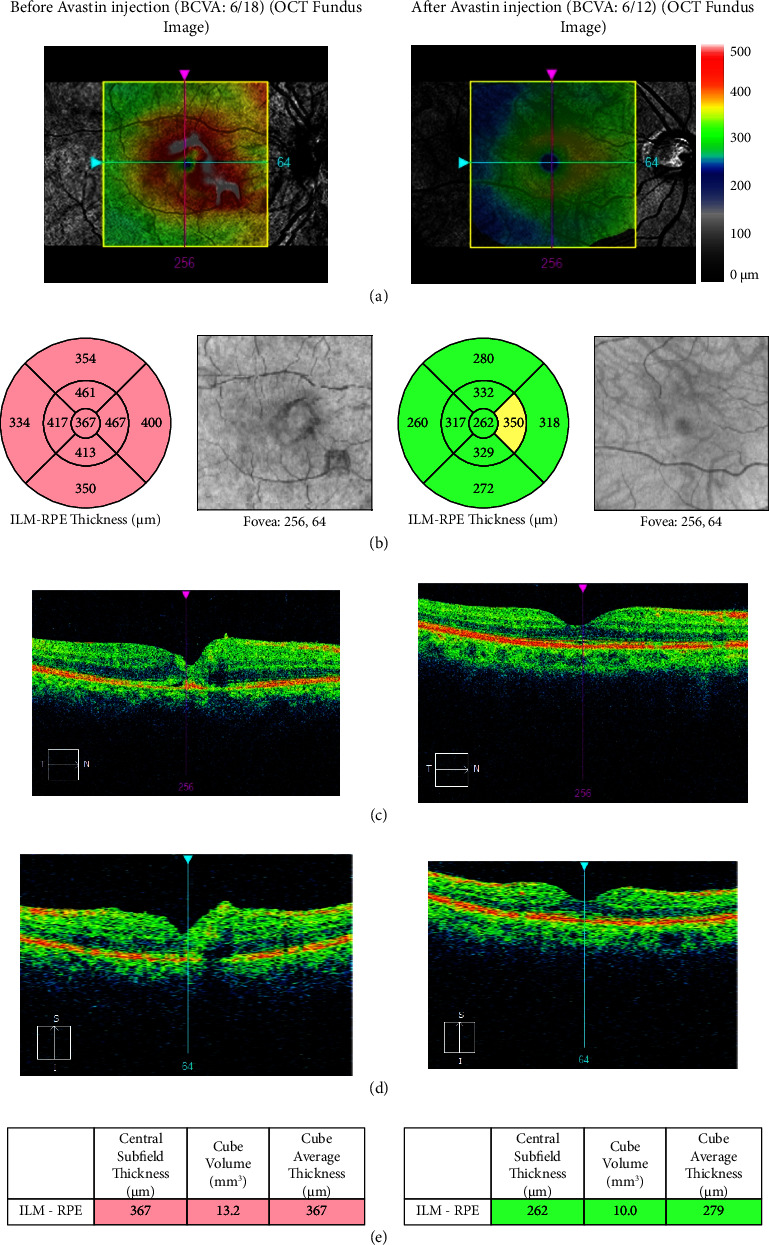
The visual outcome of NPDR (right eye) patient number 19 based on the OCT image; (a) CFP image before (left side) and after (right side); (b) OCT red free fundus image with a thickness map before (left side) and after (right side); (c) ETDRS grid with thickness measurements displayed in each macular sector before (left side) and after (right side); (d) vertical tomogram before (left side) and after (right side); and (e) value table before (left side) and after avastin injection (right side).

**Figure 8:**
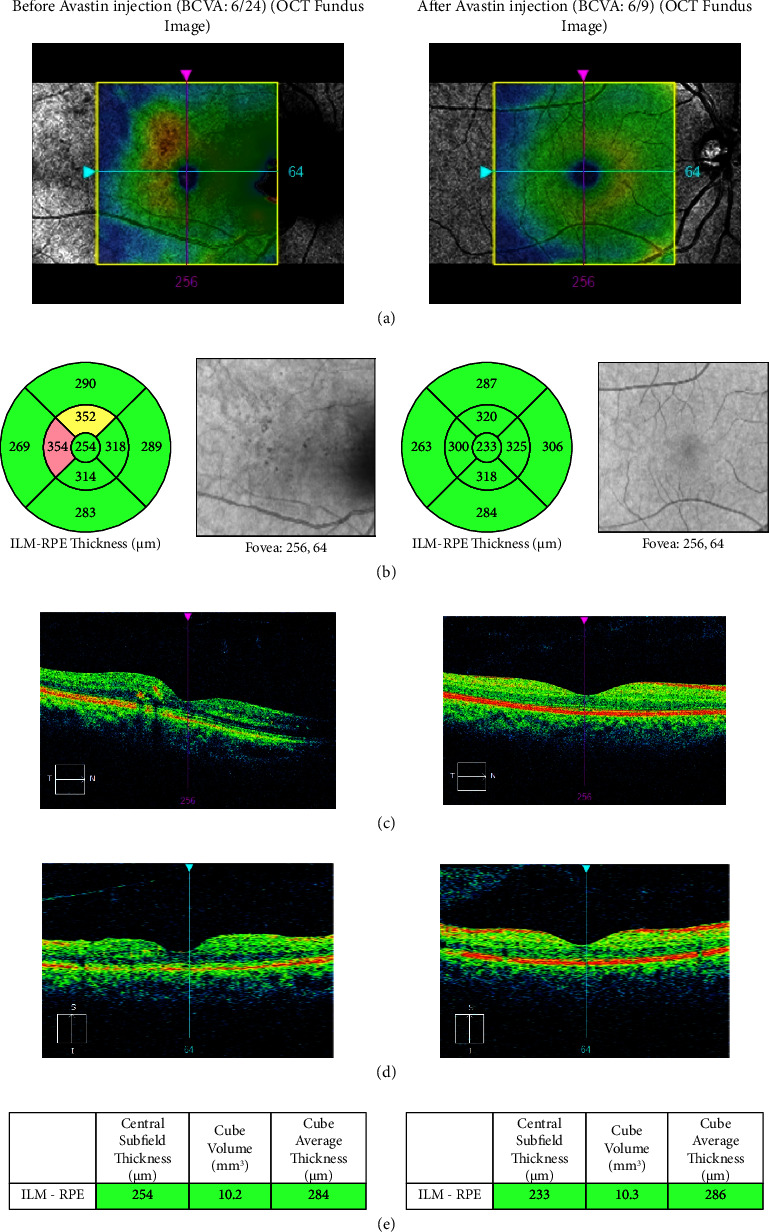
The visual outcome of NPDR (right eye) patient number 36 based on the OCT image; (a) CFP image before (left side) and after (right side); (b) OCT red free fundus image with a thickness map before (left side) and after (right side); (c) ETDRS grid with thickness measurements displayed in each macular sector before (left side) and after (right side); (d) vertical tomogram before (left side) and after (right side); and (e) value table before (left side) and after avastin injection (right side).

The OCT image and visual result for patients 19 and 36 are displayed in Figures 7 and 8. We looked closely at how the visual results of NPDR in both cases were subtly improved after Avastin injection. Prior to receiving Avastin injection, patient number 19's central subfield thickness and cube volume measured in an OCT picture were 367 *μ*m and 13.2 mm^3^, respectively. Afterward, they changed to 262 *μ*m and 10.0 mm^3^, respectively. Conversely, the OCT image's central subfield thickness and cube volume were 254 *μ*m and 10.2 mm^3^ prior to Avastin injection, respectively, and changed to 233 *μ*m and 10.3 mm^3^ for patient number 36.

## 5. Discussion

This research was an observational cross-sectional study on type 2 diabetic mellitus at Khulna BNSB Eye Hospital, Khulna, Bangladesh. The primary goal was to perform PDR and NPDR ocular examinations with CSME for individuals with type 2 diabetes who had received an Avastin injection and had BCVA and OCT measurements three months prior. Diabetic retinopathy patients have visual loss due to retinal detachment (R.D.), macular edema, and vitreous haemorrhage. The development of new vessels in the retina as a result of erythropoietin, fibroblast growth factor, endothelial growth factor, and insulin-like growth factor-1 (VEGF) is another important component contributing to DR in patients. The formation of new vessels leads to vision loss and vitreous bleeding. Patients with diabetic retinopathy experience dispersed or blurred vision, which is the result of diabetic macular edema, as well as central vision loss. However, clinically significant macular edema (CSME) is defined as 500 um of the fovea centre retinal thickness and exudates. A clinical diagnosis of macular edema can be made using OCT and CFP equipment.

**Figure 9:**
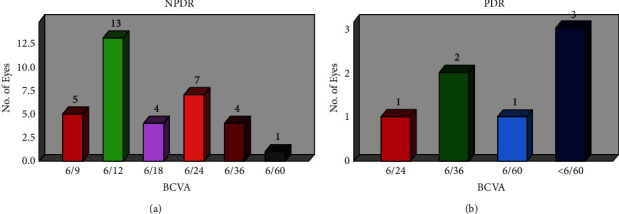
Best-corrected visual acuity (BCVA) status for (a) NPDR and (b) PDR patients' eyes after three Avastin injections.

Figure 9(a) shows the comparison of the number of eyes versus BCVA for NPDR patients with macular edema. We noticed that, following three months of Avastin injection surveillance, a sizable percentage of patients—six (6) out of thirteen eyes (13)—had BCVA. In contrast to 6/9 for five eyes, 6/18 and 6/36 for four (4) eyes, and 6/60 for the final eye, the second-highest BCVA for NPDR with macular edema was 6/24 for seven (7) eyes. However, three eyes with BCVA less than 6/60 in PDR with macular edema in Figure 7(b) represent a larger number of PDR patients than two eyes with 6/36, one eye with 6/24, and one eye with 6/60.

**Figure 10:**
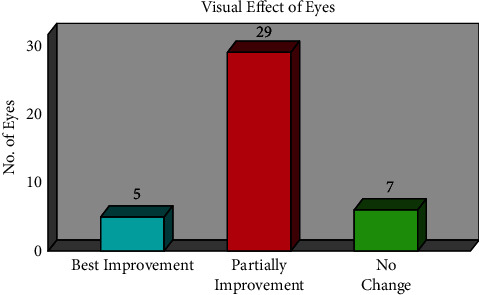
Visual improvement of both PDR and NPDR patients.

When an intravitreal Avastin injection was administered for three months, Figure 10 illustrates the visual improvement by BCVA in PDR and NPDR patients with macular edema. The study's overall findings showed that the macular edema in five of the eyes was completely resolved. The BCVA was 6/12 to 6/60 for the 29 eyes, which is referred to as partial resolution (or improvement of visual acuity somewhat). We discovered that the vision in seven eyes remained the same both before and after the Avastin injection. The effects of the Avastin injection both before and after did not alter the macular edema in seven of the eyes.

In studies of the literature, as indicated in Table 13, Mahat et al. [21] investigated the eyes of 60 individuals 48 weeks after administering a bevacizumab injection. 50 eyes had improved, 33 eyes were steady, and 4 eyes showed no improvement as a result of the clinical experiment. According to Arevalo et al. [23], 43 eyes appear to have better vision, and 32 eyes maintained a stable condition following BCVA. Additionally, Nagasawa et al. [24] examined 54 patients' eyes. Following the patient's injection of bevacizumab, the results indicated that 43 eyes had improved, 10 eyes had stable vision, and 1 eye had not significantly improved. In a retrospective study conducted by Joshi et al. [25], 53 patients' 74 eyes were investigated. According to their research, there has been an improvement in 16 eyes, a stable number of 43 eyes, and no improvement in 15 eyes related to diabetic macular edema. Thirty eyes of twenty-eight DME patients participated in a cross-sectional study by Vader et al. [26] over a period of 5.26 ± 2.39 months, with a three-month follow-up interval. It has been demonstrated that the BCVA of 12 eyes improved while that of 15 eyes remained unchanged. When compared to macular photocoagulation, Soheilian et al. [22] have shown that patients' eyes improve with DME after 24 weeks.

The research we conducted had certain limitations.

- The study period was rather brief due to anatomical responses and follow-up times
- Patients' lack of awareness caused them to respond slowly
- The visual outcome impact occasionally reveals unsatisfactory findings for people with type 2 diabetes mellitus who do not have adequate glucose control
- Sometimes, instead of using the standard ETDRS chart to measure BCVA, people use the Snellen chart
- There are further choices for treating diabetic macular edema, such as ranibizumab and aflibercept, but their costs and benefits are relatively high for the purpose of this research

## 6. Conclusion

The most prevalent cause of diabetic macular edema, which results in loss of central vision, is nonproliferative diabetic retinopathy (NPDR) or proliferative diabetic retinopathy (PDR). This condition is also the most common cause of visual impairment. Without supported grid laser photocoagulation, intravitreal Avastin injection is essential, and prompt therapy would enhance the prognosis of visual results in diabetic macular edema. Therefore, the study clearly demonstrates that intravitreal Avastin injection significantly improves BCVA in individuals with diabetic retinopathy and macular edema both before and after injection of Bevacizumab (Avastin). Based on observations made throughout the writing of this paper, the following topics were deemed deserving of further investigation:

- The study (ETDRS) has received classification
- The connection between anaemia and diabetic retinopathy in people with type 2 diabetes
- Patients with type 2 diabetes mellitus are aware of diabetic retinopathy

## Figures and Tables

**Table 1:** Best-corrected visual acuity (BCVA) of patients before Avastin injection for NPDR patients before diagnosis.

BCVA (Snell chart)	No. of eyes (*n* = 34%)
<6/60	2 (5.88)
6/60	3 (8.81)
6/36	11 (32.36)
6/24	11 (32.36)
6/18	7 (20.59)

**Table 2:** Groups of the patient according to BCVA for NPDR patients before diagnosis.

Group	No. of eyes (*n* = 34) (%)	BCVA
1	5 (14.7)	≤6/60
2	22 (64.71)	6/36 − 6/24
3	7 (20.59)	6/18

**Table 3:** Comparisons of BCVA before and after three months of Avastin treatment (NPDR patients) after diagnosis.

Group	Before Avastin injection no. of patients (*n* = 34)	3 months after the Avastin injection		*P* value
		Snell chart	No. of patients	
1	6/60 or less (*n* = 5)	6/9	1	*P* < 0.0001
		6/12–6/18	1	
		6/24–6/36	2	
		No improvement	1	

2	6/36 − 6/24 (*n* = 22)	6/6–6/9	2	*P* < 0.0001
		6/12–6/18	11	
		6/24	7	
		No improvement	2	

3	6/18 (*n* = 7)	6/6	0	*P* < 0.0001
		6/9	2	
		6/12	4	
		No improvement	1	

**Table 4:** Characteristics of patients with macular edema associated with NPDR (right eye) 16 eyes after diagnosis.

Patient no.	Baseline BCVA by Snell chart (before 3 months)	Baseline macular thickness by OCT (mm)	Final BCVA by Snell chart (after 3 months)	Final macular thickness by OCT (mm)
5	6/60	308	6/36	303
8	6/36	375	6/24	267
10	**6/60**	**479**	**6/60**	**454**
19	6/18	367	6/12	262
24	6/24	335	6/12	259
28	6/36	358	6/24	247
29	6/18	310	6/12	261
35	6/36	327	6/18	277
36	6/24	254	6/9	233
39	6/36	314	6/36	285
42	6/24	302	6/12	252
43	**6/36**	**429**	**6/18**	**267**
56	6/36	378	6/24	259
57	6/36	410	6/24	358
58	6/24	283	6/12	251
70	6/60	436	6/18	284

All the bold patient's conditions as stated bold values.

**Table 5:** Characteristics of patients with macular edema associated with NPDR (left eye) 18 eyes after diagnosis.

Patient no.	Baseline BCVA by Snell chart (before three (3) months)	Baseline macular thickness by OCT (mm)	Final BCVA by Snell chart (after three (3) months)	Final macular thickness by OCT (mm)
1	<6/60	415	6/36	361
8	6/36	358	6/24	328
10	6/18	345	6/18	273
22	6/36	437	6/24	252
24	6/24	361	6/12	266
28	6/36	462	6/24	298
29	6/18	306	6/12	252
35	6/24	358	6/12	278
36	**6/24**	**317**	**6/9**	**221**
42	6/24	366	6/12	253
43	**6/60**	**459**	**6/9**	**218**
50	6/18	283	6/12	264
56	6/24	341	6/12	272
57	**6/18**	**364**	**6/9**	**220**
58	6/24	295	6/12	259
59	6/24	291	6/12	264
60	6/36	357	6/36	338
88	6/18	276	6/9	217

All the bold patient's conditions as stated bold values.

**Table 6:** Outcome on edema resolution for NPDR patients after diagnosis.

Outcome of Avastin	No. of eyes (*n* = 34%)
Edema completely resolved	5 (14.7)
Edema partially resolved	25 (73.53)
Edema not resolved	4 (11.77)

**Table 7:** BCVA of patients before injection of Avastin for PDR patients after diagnosis.

BCVA by Snell chart	No. of eyes (*n* = 7) (%)
<6/60	5 (71.42)
6/60	2 (28.58)

**Table 8:** Groups of the subject according to BCVA (PDR patients).

Group	BCVA	Number of eyes (*n* = 7) (%)
1	<6/60	5 (71.42)
2	6/60	2 (28.58)

**Table 9:** Comparisons of BCVA in groups before and after three (3) months of Avastin treatment for PDR patients after diagnosis.

Group	Before Avastin injection (no. of eyes (*n* = 7))	3 month after the Avastin injection		*P* value
		Snell chart	No. of patients *n* (%)	
1	<6/60 (*n* = 5)	6/6–6/9	0 (0.0)	*P* < 0.0001
		6/12–6/24	0 (0.0)	
		6/36	1 (20.0)	
		No improvement	4(80.0)	

2	6/60 (*n* = 2)	6/6–6/18	0 (0.0)	*P* < 0.0001
		6/24	1 (50.0)	
		6/36	1 (50.0)	
		No improvement	0 (0.0)	

**Table 10:** Characteristics of patients with macular edema associated with PDR (right eye) after diagnosis.

Patient no.	Baseline BCVA by Snell chart (before three (3) months)	Baseline macular thickness by OCT (mm)	Final BCVA by Snell chart (after three (3) months)	Final macular thickness by OCT (mm)
23	6/60	363	6/24	279
32	<6/60	472	6/60	384
33	<6/60	468	<6/60	456

**Table 11:** Characteristics of patients with macular edema associated with PDR (left eye) after diagnosis.

Patient no.	Baseline BCVA by Snell chart (before three (3) months)	Baseline macular thickness by OCT (mm)	Final BCVA by Snell chart (after three (3) months)	Final macular thickness by OCT (mm)
5	6/60	381	6/60	391
23	<6/60	326	6/36	273
32	<6/60	453	<6/60	390
33	<6/60	573	<6/60	471

**Table 12:** Outcome of Avastin on edema resolution for PDR patients after diagnosis.

The outcome of Avastin injection	Number of eyes (*n* = 7) (%)
Edema completely resolved	0 (0.0)
Edema partially resolved	3 (42.85)
Edema not resolved	4 (57.15)

**Table 13:** Comparative study of this research work with state of the art research work.

Ref	Study period	Study of population (DM with DR)	Disease type	Injection of Bevacizumab (Avastin)	Improve no. of eyes (%)	Stable no. of eyes (%)	No improvement no. of eyes (%)	Follow-up periods
Maha et al. [21]	2016	87 eyes (60 patients)	DME	Yes	57.47%(50 eyes)	37.94% (33 eyes)	4.6% (4 eyes)	48 weeks
Arevalo et al. [23]	2005	75 eyes	PDR	Yes	57.3% (43 eyes)	42.66% (32 eyes)	0	6.31 ± 0.81 months
Nagasawa et al. [24]	2011	54 eyes (54 patients)	DME	Yes	79.6% (43 eyes)	18.5% (10 eyes)	1.9% (1 eye)	6 month
Joshi et al. [25]	2011	78 eyes (54 patients)	DME	Yes	23% (18 eyes)	56% (43 eyes)	21% (17 eyes)	12–34 months
Vader et al. [26]	2008	30 eyes (28 patients)	DME	Yes	40% (12 eyes)	50% (15 eyes)	10% (3 eyes)	5.26 ± 2.39 months
Soheilian et al. [22]	2008	150 eyes (129 patients)	DME	Yes	37% (56 eyes)	25% (37 eyes)	14.8% (22 eyes)	36 weeks
This research work	2022	41 eyes (25 patients)	PDR and NPDR with CSME	Yes	82.92% (34 eyes)	17.1% (7 eyes)	0 (0 eyes)	1 year

## Data Availability

The data used to support this study are available from the corresponding author on request.

## Additional Points

*Synopsis*. This work is based on actual examination studies of people with diabetic retinopathy. Diabetes patients with macular edema in their eyes were gathered in 2021, and Bevacizumab injections were administered along with BCVA eye exams. These trials were conducted for three, six, nine, or one year, depending on the state of the eyes. Later, we examined each eye using an OCT scan and CFP to see whether there had been any progress.
